# Rotationally Resolved Predissociation Spectrum of the 1^5^Φ ← X^5^Δ Rovibronic Band of FeH^+^

**DOI:** 10.1021/acs.jpclett.5c03818

**Published:** 2026-01-28

**Authors:** Shan Jin, Marc Reimann, Christian van der Linde, Milan Ončák, Martin K. Beyer

**Affiliations:** Universität Innsbruck, 27255 Institut für Ionenphysik und Angewandte Physik, Technikerstraße 25, 6020 Innsbruck, Austria

## Abstract

The elusive diatomic molecule FeH^+^ has long been hypothesized to exist in cool interstellar environments, yet its spectral signature has remained unidentified due to the lack of laboratory data. Its neutral counterpart FeH, on the other hand, is a well-known feature in the atmospheres of M-dwarfs, Sunspots, and hot Jupiter. Here we present the first rotationally resolved photodissociation spectrum of gas-phase FeH^+^, covering the energy range of 18550–18830 cm^–1^ (5390.8–5310.7 Å). The rotational structure of the 1^5^Φ ← X^5^Δ electronic transition is resolved and conclusively assigned using state-of-the-art multireference and coupled cluster calculations and rovibrational spectra simulations. FeH^+^ spectral peaks in this specific band are broadened by predissociation, which arises from curve crossings of the bound 1^5^Φ potential curve with those of repulsive ^7^Π, ^7^Δ states. The photodissociation cross-section does not exceed 5 × 10^–20^ cm^2^, and comparison of the laboratory spectrum with observation data from HD 183143 does not reveal a match for this relatively weak band. Due to the uncertainty of the spin–rotation coupling constant, it is not possible to predict the precise positions of the rotational line spectrum. However, the high spin and orbital angular momentum quantum numbers of the electronic ground state place the onset of the spectrum above 1300 GHz, a frequency region that cannot be observed with ground-based telescopes.

## Graphical abstract

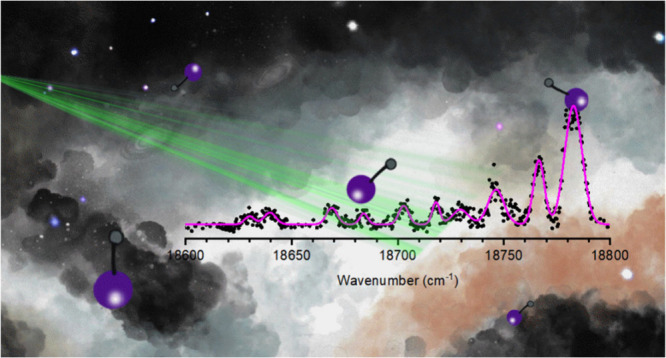

Iron is one of the most abundant metals in the universe. Since only on the order of 1% of the expected iron abundance is found in the form of neutral or ionized atoms in the interstellar medium (ISM),^1^ nanoparticles^2^ and iron pseudocarbynes^3^ have been proposed as possible sinks. Detections of FeO in Sgr B2 ^4^ and FeCN in IRC+10216 ^5^ suggest that molecular iron species occur in diverse environments, from the ISM near the Galactic center to circumstellar envelopes. Among potential iron-bearing molecules, FeH^+^ is a prime candidate: it is ionized, like most of the atomic iron inventory in the ISM, and hydrogen is the by far most abundant element. It therefore has been proposed for decades to exist in the cool ISM.^6,7^ Quantum chemical analyses of its electronic and geometric structure^8–15^ as well as its formation in ion–molecule reactions at elevated kinetic energies^16–19^ are well established. We recently provided the first laboratory infrared spectra of FeH^+^ by argon tagging infrared multiple photon dissociation (IRMPD) spectroscopy of the fundamental and overtone of the Fe–H stretching mode.^20,21^ Recently, we also obtained X-ray absorption data in collaboration with the group of Lau at the BESSY II synchrotron radiation source.^22^

For identification in the ISM, however, high-resolution laboratory spectra of FeH^+^ without tagging are required in a spectral region that is covered by equally high-resolution astronomical observations. Although Langhoff and Bauschlicher^12^ found shallow potential minima in the potential curves of excited quintet states of FeH^+^, they concluded that the Franck–Condon factors of the transitions are too small, so that the absorption spectrum of FeH^+^ is expected to consist of broad, structureless bands arising from excitation into repulsive parts of the potential curves. In contrast to these expectations, we here report a rotationally resolved photodissociation spectrum of a 1^5^Φ ← X^5^Δ transition of FeH^+^ in the optical region. Observation of these transitions in a photodissociation spectrum is made possible by predissociation via repulsive septet states, which also explains the observed line broadening.

The molecular cation FeH^+^ is produced using a standard laser vaporization source,^23–25^ and trapped in a liquid-nitrogen cooled ion cyclotron resonance (ICR) cell^26^ at ca. 80 K. The tunable light is generated by an optical parametric oscillator (OPO) system. Figure 1 displays a rotationally resolved rovibronic band of FeH^+^ in the region of 18550–18830 cm^–1^, along with Gaussian fits to the individual peaks. Peak positions and widths are listed in Table 1. One should notice that the band width values reported in Table 1 represent the experimentally observed widths, which include a contribution from the OPO line width (<3 cm^–1^). The most intense peak is centered at 18783 cm^–1^, followed by a series toward lower energy with decreasing intensity. The photodissociation cross sections σ of all peaks are below 5 × 10^–20^ cm^2^. The peak width (full width at half-maximum, fwhm) determined from the Gaussian fits are in the range of 4–10 cm^–1^. However, some peaks are quite blurred, almost merging with the detection limit.

**Figure 1:**
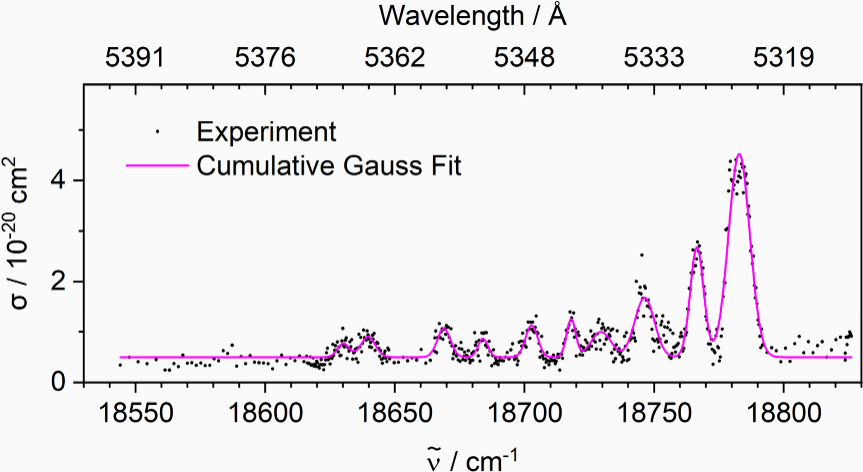
Photodissociation spectrum of FeH^+^ with Gaussian fits to rotational lines. A baseline of 5 × 10^–21^ cm^2^ was chosen, which we attribute to incomplete removal of the very intense Fe^+^ signal during mass selection.

**Table 1:** Peak Positions *ν̃* Extracted from Figure 1 Together with the Full Width at Half-Maximum (fwhm) and Peak Assignments from the Empirical and AQC Fits for Comparison

Gaussian fit			empirical fit		AQC fit	
*ν̃*/cm^–1^	fwhm/cm^–1^	*ν̃*/Å	*ν̃*/cm^–1^	*J*′ ← *J*″	*ν̃*/cm^–1^	*J*′ ← *J*″
18783	9.70	5324.0	18783	5 ← 4	18783	5 ← 4
18766	6.09	5328.8	18766	6 ← 5	18766	6 ← 5
18746	8.84	5334.5	18745	7 ← 6	18745	7 ← 6
18729	9.10	5339.3	18731	5 ← 5	18728	5 ← 5
18718	4.14	5342.5	18718	8 ← 7	18720	8 ← 7
18703	5.52	5346.7	18703	6 ← 6	18700	6 ← 6
18684	4.48	5352.2	18687	9 ← 8	18689	9 ← 8
18669	6.03	5356.5	18671	7 ← 7	18667	7 ← 7
18640	6.58	5364.8	18650	10 ← 9	18645	6 ← 5^a^
18630	5.43	5367.7	18633	8 ← 8	18630	8 ← 8

a The only line with (*J* – *N*) = 1; all other lines (*J* – *N*) = 2, with nuclear rotational quantum number *N*.

Figure 2 presents the quintet and septet potential energy curves for FeH^+^ that connect to the H­(^2^S) + Fe^+^(^4^F) and H­(^2^S) + Fe^+^(^6^D) asymptotes. The complete scan is shown in Supporting Information Figure S1. The shaded areas in Figure 2 indicate the range where the potential curves calculated with spin–orbit coupling are found. The excited quintet states (blue) of FeH^+^ lie ca. 18000–20000 cm^–1^ above the X^5^Δ ground state minimum. These excited state potential curves exhibit very shallow minima, implying a weakly bound state with a long Fe–H bond. Transitions from the ground state into three of these states, namely 1^5^Φ, 1^5^Π, and 1^5^Δ, are fully allowed under the selection rules for electric dipole transitions, while the Franck–Condon overlap is limited.

**Figure 2:**
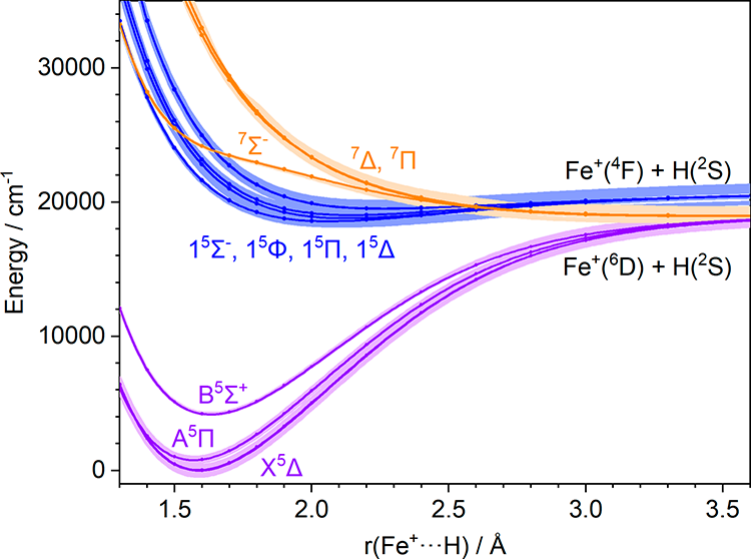
Potential energy curves of quintet (purple) and septet (yellow) states of FeH^+^ correlating with the H­(^2^S) + Fe^+^(^6^D) asymptote as well as quintet states (blue) correlating with the H­(^2^S) + Fe^+^(^4^F) asymptote. Shaded areas denote the range of spin–orbit splitting. Calculated at the MRCI+Q­(8,12)/aug-cc-pVQZ level of theory.

To learn more about the nature of the observed rovibronic band, we simulated the rotational structure of the electronic transitions 1^5^Φ ← X^5^Δ (ΔΛ = +1), 1^5^Π ← X^5^Δ (ΔΛ = −1), and 1^5^Δ ← X^5^Δ (ΔΛ = 0) using PGOPHER.^27^ Due to the relatively small number of lines, their width and the large number of spectroscopic constants that determine the peak positions and intensities, the fit algorithm of PGOPHER did not converge to physically reasonable results. In order to obtain meaningful simulated spectra by manually adjusting the parameters, we had to reduce the parameter space. To this end, we estimated the rotational constants of ground and excited state from the curves in Figure 2. All other parameters were obtained empirically by fitting the experimental bands. Initial guesses for the spin–orbit coupling constants were estimated from the respective elements of the spin–orbit matrix. These empirical fits are shown in Figure S2 of the Supporting Information, the respective parameters are listed in Table S1, and the best match is shown in Figure 3a. In all fits, we neglected lambda doubling, as the effect is typically in the order of ∼0.01 cm^–1^ and thus well below our observed line widths.

**Figure 3:**
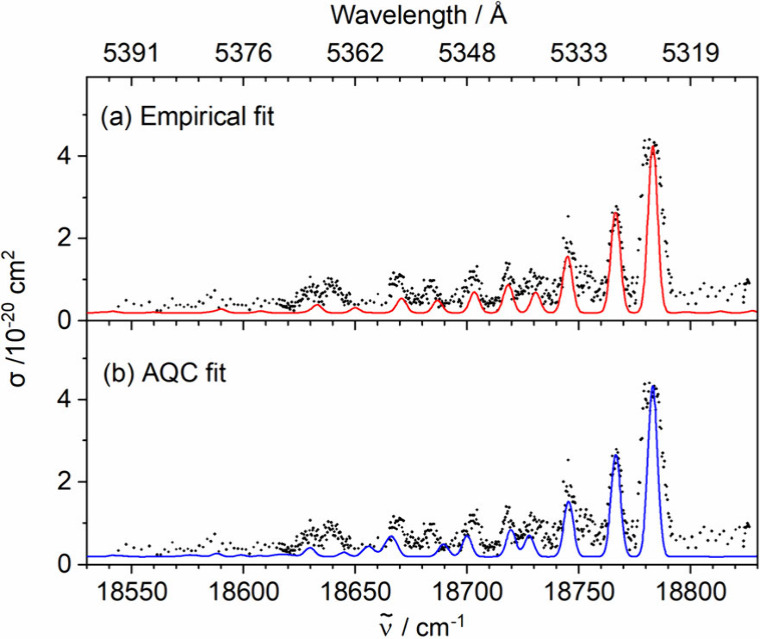
Simulations of the rovibronic structure for the 1^5^Φ ← X^5^Δ transition using (a) the initial empirical fit and (b) the refined AQC set of parameters described in the text. Experimental peak positions and simulation assignments are provided in Table 1. The experimental spectrum is included for reference.

Only the 1^5^Φ ← X^5^Δ transition reproduces the relative intensities of the rovibronic peaks in the experimental spectrum. In contrast, 1^5^Π ← X^5^Δ and 1^5^Δ ← X^5^Δ transitions exhibit an additional peak on the high-energy side of the most intense features, with this peak being the third-/fourth-most intense one. We therefore assign our observed transition as 1^5^Φ ← X^5^Δ. Based on this assignment, we tried to refine our fitting parameters using quantum chemical calculations. As even high-level calculations do typically not reach spectroscopic accuracy for transition metal systems, we only used the calculated values as starting point for a second fit of the experimental spectrum. The obtained parameter set (labeled “adjusted quantum chemical”, AQC) is shown in Table S2, the two resulting fits are shown in Figure 3. While both sets of parameters (see Tables S1 and S2) differ quite significantly, we cannot clearly assign one set or the other, due to the limited resolution and number of lines of our experimental spectrum. The rotational quantum numbers of initial and final state in Table 1 show that only the R- and Q-branch of the 1^5^Φ ← X^5^Δ electronic transition are experimentally observed. The fits yield a negative spin–orbit coupling constant, which is also predicted by quantum chemistry, which results in an Ω = 4 ground state for a Hund’s case a. Consequently, the rovibronic lines start with *J*″ = 4.

Our calculations suggest that several vibrational states (*v*′ < 5) of the 1^5^Φ state could be the target state of our transition, where higher values of *v*′ have higher Franck–Condon overlap with the vibronic ground state. Based on the width of our experimental spectrum and the calculated vibrational frequency of the excited state of about 525 cm^–1^, we assume that we only observe a single value for *v*′. However, the specific value of *v*′ depends on the crossing-point of the quintet and the dissociative septet curve needed for predissociation. An assignment based on the experimental spectrum is impossible due to the small number of observed lines and the line broadening caused by predissociation. Quantum chemistry is also not able to resolve the issue with confidence: the position of the crossing point is very sensitive to the relative energy of the relevant spin–orbit component of the septet state and the curvature of the 1^5^Φ state. Even state-of-the-art computational methods such as MRCI are simply not precise enough to give an unambiguous answer.

While both the X^5^Δ ground state and the 1^5^Φ excited state have multireference character, our MRCI calculations show them to be strongly dominated by σ^2^δ^3^π^2^σ*^1^ and σ^1^δ^3^π^3^σ*^1^ configurations, respectively. A qualitative representation of the involved orbitals, which are all strongly dominated by iron-centered atomic orbitals, is shown in Figure S5. The observed transition can therefore be classified as a σ → π transition. Due to the largely nonbonding character of the iron d-orbitals of π symmetry in the FeH^+^ molecular ion, this leads to a reduction of the bond order and thus explains the increase in bond length in the excited state.

This increased bond length in the excited state leads to a poor Franck–Condon overlap between ground and excited state, which further reduces the absorption cross section on top of the, according to the calculations, small electronic transition moment.

This study provides the first rotationally resolved spectrum of FeH^+^ in a spectral range that could be suitable for its identification in astronomical data. Unassigned features in UV/vis spectra of the interstellar medium are the famous diffuse interstellar bands (DIBs), first reported in 1922,^28^ one of the most compelling mysteries in astrophysics.^29,30^ Five DIBs have been assigned to C_60_^+^ via helium tagging spectroscopy^31,32^ and by Hubble space telescope observations.^33^ A comprehensive survey of DIBs in the 3800–8680 Å range by Jenniskens et al.^34^ concluded that most bands exhibit narrow widths – typically subwavenumber (≤1 cm^–1^) or a few wavenumbers (1–5 cm^–1^). E.g., the confirmed DIBs carried by C_60_^+^ have bandwidth less than 3 cm^–1^.^31,32,35^ The experimental peaks of FeH^+^ observed in this study are located near two known DIBs at 5362.14 Å (18644 cm^–1^) originally reported by Herbig^36^ and 5363.60 Å (18639 cm^–1^) originally reported by Jenniskens et al.^34^ and later included in the comprehensive DIB catalog by Hobbs et al. obtained with the 3.5 m telescope and the ARC echelle spectrograph (ARCES) at Apache Point Observatory.^37^ Additionally, observational data reveal Fe II and S II atomic absorption lines at 5316.65 Å and 5320.73 Å,^38^ as well as several unassigned absorption features. However, none of these features match the FeH^+^ peaks observed in the laboratory. Figure S3 shows a comparison of our laboratory spectrum with the spectrum of HD 183143. The lack of a match shows that FeH^+^ is not the carrier of these specific DIBs. The Fe II absorption at 5317 Å occurs in the stellar atmosphere. The lack of any trace of the FeH^+^ absorption shows that the column density of FeH^+^ in the ISM along this sightline is smaller than the detection limit. The relatively low absorption cross section of FeH^+^ highlights the challenges in detecting FeH^+^ in space.

The fit parameters from Tables S1 and S2 allow in principle to predict the pure rotational spectrum, within the uncertainties of the fit parameters. To check if this is a viable route toward detection, we simulated two rotational spectra of FeH^+^, using the two sets of fit parameters derived in this work, as shown in Figure S4. Obviously, the parameters are not yet sufficiently accurate for this purpose, with peak positions shifting by more than 100 GHz. The biggest unknowns are the spin–spin coupling constant λ and the spin-rotation coupling constant γ, for which no reliable computational protocols exist. To refine the parameters and enable comparisons with astronomical data, future work should focus on rotationally resolved measurements of additional bands which are not broadened by predissociation, which requires different experimental techniques, e.g., two-color resonance enhanced photodissociation. This technique can also be employed for the study of rovibrational transitions. The gold standard, of course, would be a direct measurement of the rotational spectrum in the laboratory. A two-color detection scheme for THz spectroscopy of rotational transitions might be based on the depletion or enhancement of rovibronic lines in our spectrum reported in Figure 1.

We provide the first rotationally resolved photodissociation spectrum of FeH^+^. The 1^5^Φ ← X^5^Δ band was observed in the region of 18550–18830 cm^–1^ by recording the Fe^+^ fragment signal, which arises from predissociation via one or more repulsive septet states. High-level quantum chemical calculations and rovibrational spectra simulations provide a consistent picture, reproducing the rotationally resolved spectrum with the most intense peak for the 1^5^Φ (*J*′ = 5) ← X^5^Δ (*J*″ = 4) transition at 18783 cm^–1^. Using our experimental data, we successfully simulated the rotationally resolved band shape of the 1^5^Φ ← X^5^Δ electronic transition with contributions from R- and Q-branches, while the P-branch is suppressed in the PGOPHER simulation. Understanding the electronic structure is key to the identification of FeH^+^ and to exploring its potential contribution to the chemical complexity of the ISM. With further rotationally resolved experiments with different experimental techniques, we might extract more precise spectroscopic constants to compare with astronomical data in other spectral regions, particularly in the THz regime. As a side remark, the rovibronic line broadening by predissociation underlines that diatomic molecules or molecular ions can be DIB carriers, even if they have high rotational constants.

## Experimental and Computational Methods

Experiments have been performed on a modified Bruker/Spectrospin CMS47X Fourier transform ion cyclotron resonance (FT-ICR) mass spectrometer described in previous work.^39,40^ The ions of interest were produced by an external laser vaporization ion source,^23–25^ equipped with a frequency-doubled Quantum Light Q2-D33-1053 Nd:YLF laser and an isotopically enriched iron target, ^56^Fe (99.93%, U.S. Department of Energy (DOE)). The carrier gas helium is seeded with 1% hydrogen, and is guided to the center of ICR cell, where ions can be stored and mass-selected in a 4.7 T magnetic field.^41^ The ions are cooled to low rotational temperatures during supersonic expansion. Additionally, the ICR cell is externally cooled with liquid nitrogen to ca. 80 K, to minimize the contribution of blackbody infrared radiative heating.^26,42–49^

The photodissociation spectrum is recorded by ion irradiation with a tunable OPO laser system into the ICR cell through a CaF_2_ window. Tunable monochromatic visible radiation is generated by an EKSPLA NT342B optical parametric oscillator laser system with 20 Hz pulse repetition rate, covering the experimental region with typical average laser pulse energy of 12–15 mJ. The wavelength was calibrated by a commercial echelle spectrometer (SHR high-resolution wide range spectrometer, SOLAR Laser Systems), which determined the bandwidth as <3 cm^–1^ in the experimental spectral region, consistent with the specification of <5 cm^–1^ given in the data sheet.

Loss of the hydrogen atom was the only fragmentation channel observed. Dissociation into Fe + H^+^ is 5.7 eV higher in energy and is therefore not accessible. The photodissociation cross-section σ is calculated by the modified Beer–Lambert law, eq 1.^50^

1 $$\sigma_{\mathrm{tot}}=\frac{hcA}{\lambda pEY}\ln\left(\frac{\overset{n}{\underset{i=0}{\sum }}I_{i}}{I_{0}}\right)$$

with *h* as Planck’s constant, *c* as the speed of light, *A* as the laser beam area, λ as the laser wavelength, *p* as the number of pulses, *Y* as the loss of energy due to the CaF_2_ prisms used for beam steering, *E* as the pulse energy, *I*_0_ as the intensity of the precursor ion, *I*_*i*_ (*i* > 0) as the fragment ion intensity. A major uncertainty of the cross-section is the laser beam area inside the ICR cell which is difficult to determine. The estimation of uncertainty is within a factor of 2 of the actual values. The band positions and fwhm are determined by fitting Gaussian line profiles to the experiment using Origin, with an uncertainty of 50% for the fwhm throughout the manuscript.

The potential energy curves with and without spin–orbit coupling were calculated employing multireference configuration interaction (MRCI) with the active space of 8 electrons in 12 orbitals (including the 1s orbital of H and the 3d and 4sorbitals of Fe, as well as a d double shell) and aug-cc-pVQZ basis set along with the Davidson correction, MRCI­(8,12)+Q/aug-cc-pVQZ, using Molpro.^51,52^ Parameters for the AQC fit (Table S2) were calculated at structures optimized at the X2C-MRCI+Q/aug-cc-p­(wc)­VTZ-DK level. The spin–orbit coupling (SOC) constants A were obtained directly from the corresponding SOC matrix elements between the corresponding states. The spin-rotation constants γ were obtained from *g*-tensors using the approach proposed by Curl,^53^ calculated at the QDPT level with NEVPT2 diagonal energy using the same basis set and the ORCA program package.^54^ The spin–spin coupling constants λ were obtained as half of the zero-field splitting *D*-value^55^ calculated at the PBE/QZ4P-J level using the ADF engine of the AMS program package.^56^

## Supplementary Material





## Notes

Additional computational results and comparisons with experimental findings (PDF)
Numerical data for all figures (XLSX)
