# Supplementary material for: Rotationally Resolved Predissociation Spectrum of the 15Φ ← X5Δ Rovibronic Band of FeH+
Source: J Phys Chem Lett. 2026 Jan 28;17(6):1729–34. doi: 10.1021/acs.jpclett.5c03818 (PMC12908152; doi:10.1021/acs.jpclett.5c03818)
Supplement: Supplementary file 1 [file jz5c03818_si_001.pdf]

Rotationally Resolved Predissociation Spectrum of the $1^5\Phi \leftarrow X^5\Delta$ Rovibronic Band of FeH^+

Shan Jin [a], Marc Reimann [a], Christian van der Linde [a], Milan Ončák [a*], and Martin K. Beyer [a*]

[a] *Universität Innsbruck, Institut für Ionenphysik und Angewandte Physik, Technikerstraße 25, 6020 Innsbruck, Austria*

Shan Jin	shan.jin@uibk.ac.at	0000-0002-9460-853X
Marc Reimann	Marc.Reimann@uibk.ac.at	0000-0002-3728-2983
Christian van der Linde	Christian.Van-Der-Linde@uibk.ac.at	0000-0003-0493-820X
Milan Ončák	Milan.Oncak@uibk.ac.at	0000-0002-4801-3068
Martin K. Beyer	Martin.Beyer@uibk.ac.at	0000-0001-9373-9266

Supporting Information

Contents

1. Potential energy curves of FeH^+ with Spin-orbit coupling

2. Simulated transitions for $1^5\phi$, $1^5\Pi$, and $1^5\Delta \leftarrow {}^5\Delta$ in FeH^+ using PGOPHER

3. Comparison between laboratory spectrum of FeH^+ and observations

4. Simulated pure rotational spectra of FeH^+

5. Molecular orbitals of $X^5\Delta$ and $1^5\Phi$ states

6. Parameters used for PGOPHER in Figure S2

7. AQC parameters used for PGOPHER in Figure 3b

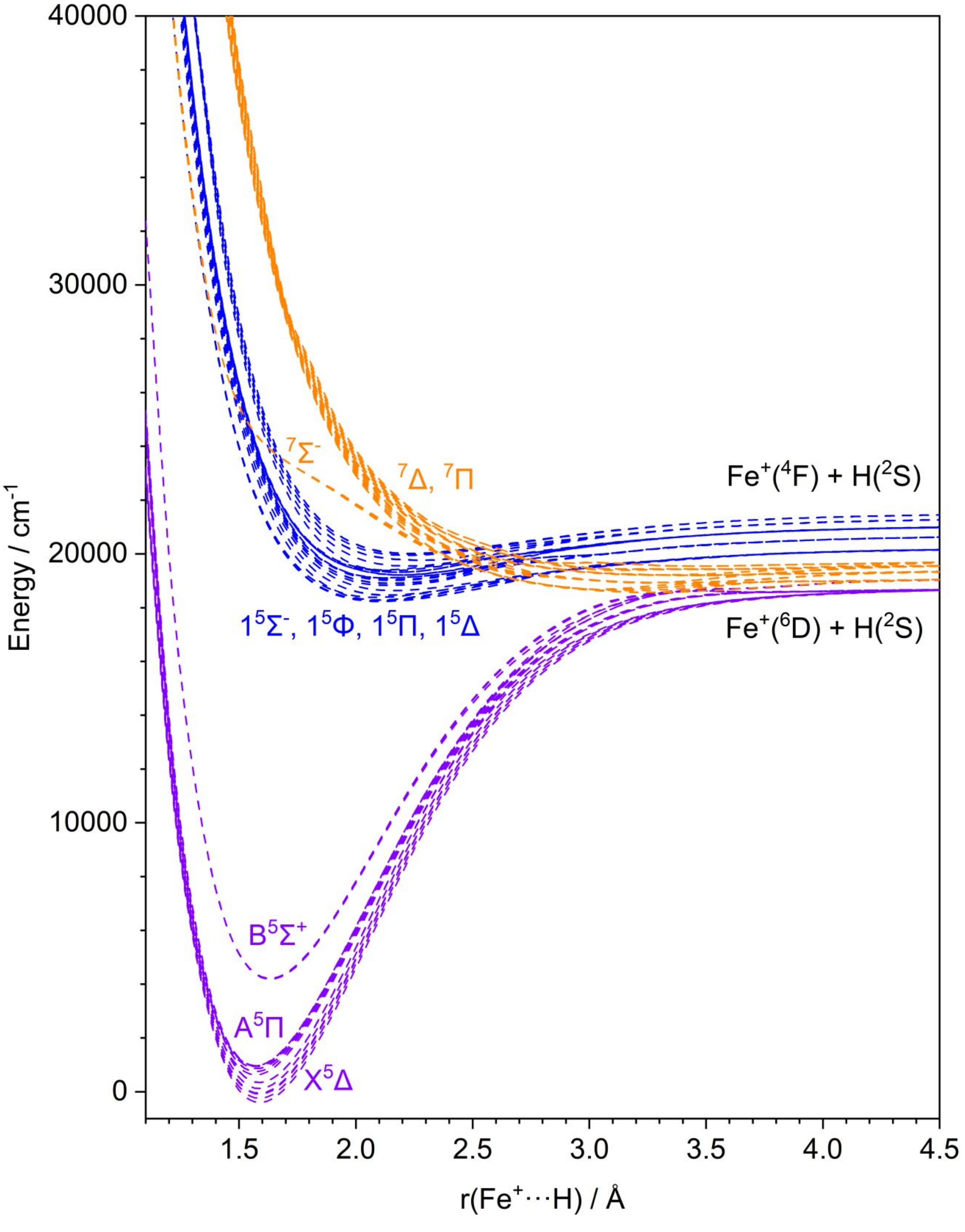

Figure S1. Potential energy curves of quintet (purple dashed lines) and septet (yellow dashed lines) states of FeH^+ correlating with the H(^{2}S) + Fe^+(^{6}D) asymptote as well as quintet states (blue dashed lines) correlating with the H(^{2}S) + Fe^+(^{4}F) asymptote, calculated at the MRCI+Q (8,12)+SOC/aug-cc-pVQZ level of theory.

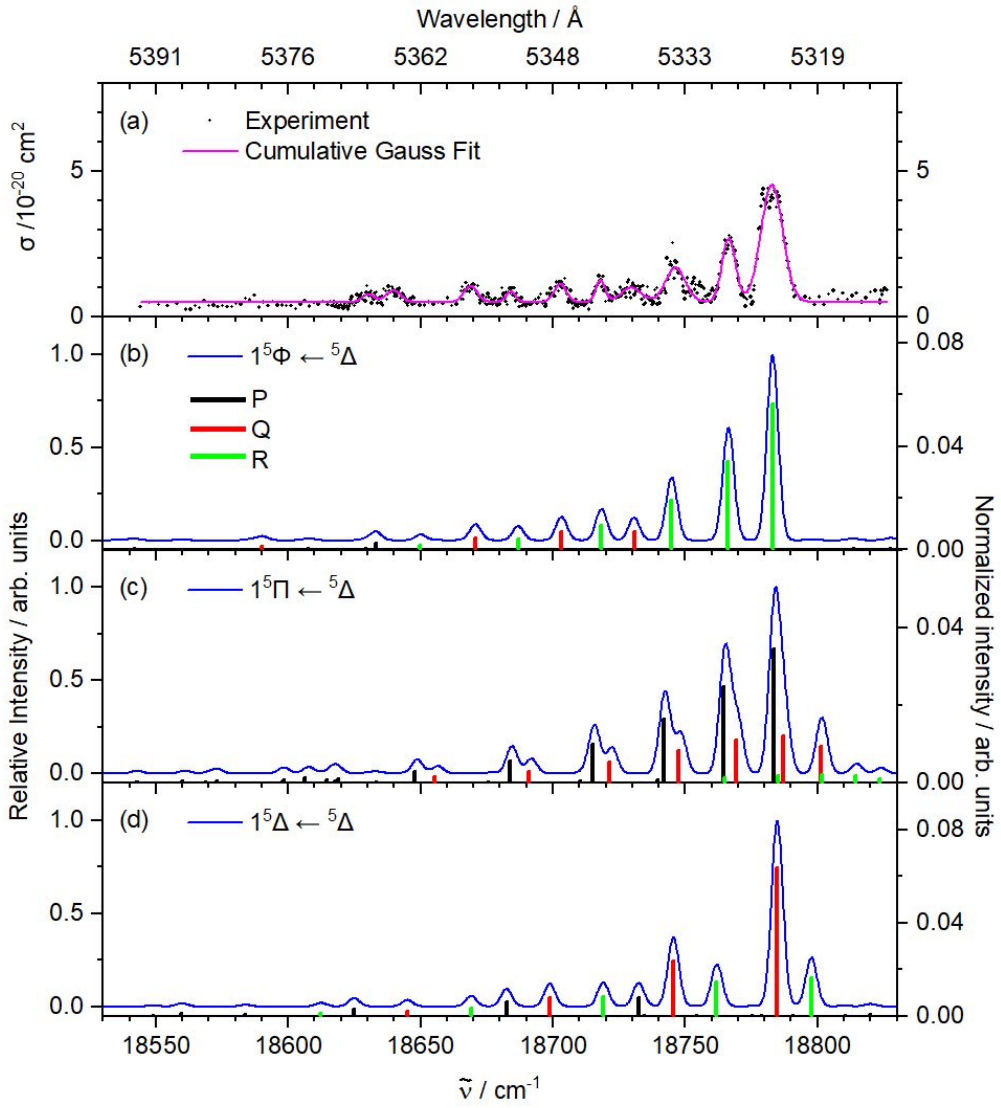

Figure S2. a) Photodissociation spectrum of FeH^+ with Gauss fits to rotational lines; simulations of the rovibronic structure assuming b) $1^5\phi \leftarrow {}^5\Delta$, c) $1^5\Pi \leftarrow {}^5\Delta$, and d) $1^5\Delta \leftarrow {}^5\Delta$ transitions in FeH^+. For all fits, the rotational constants were estimated from quantum chemical calculations and the remaining parameters were empirically adjusted to give the best agreement with the experimental spectrum.

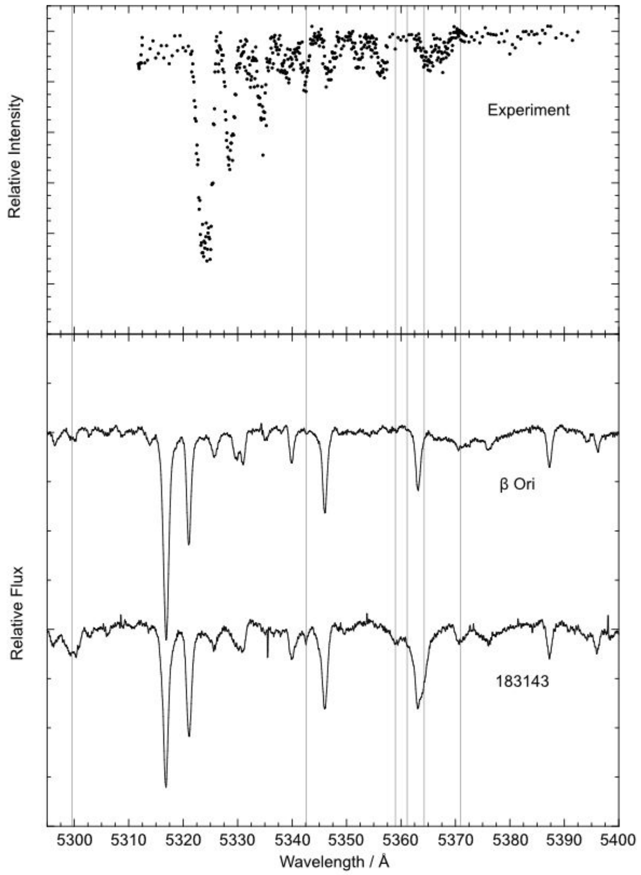

Figure S3. Comparison of the FeH^+ laboratory spectrum (upper panel) with observation data (lower panel) extracted from Hobbs et al.[1] Vertical lines indicate the unassigned DIB profiles.

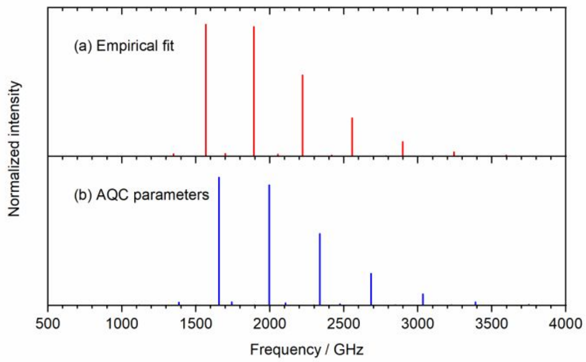

Figure S4. Simulated pure rotational spectra of FeH^+ at 100 K in its electronic ground state, generated using PGOPHER. (a) Spectrum generated using empirical fit parameters for FeH^+, derived from the $1^5\Phi \leftarrow X^5\Delta$ electronic transition shown in Figure 3a. (b) Spectrum generated using the AQC parameters for FeH^+, as detailed in Figure 3b, which exhibits a blueshift toward to higher frequencies relative to (a) the Empirical fit. The lines are subject to Lambda doubling, but the splitting is very small and not resolved on the scale of the figure.

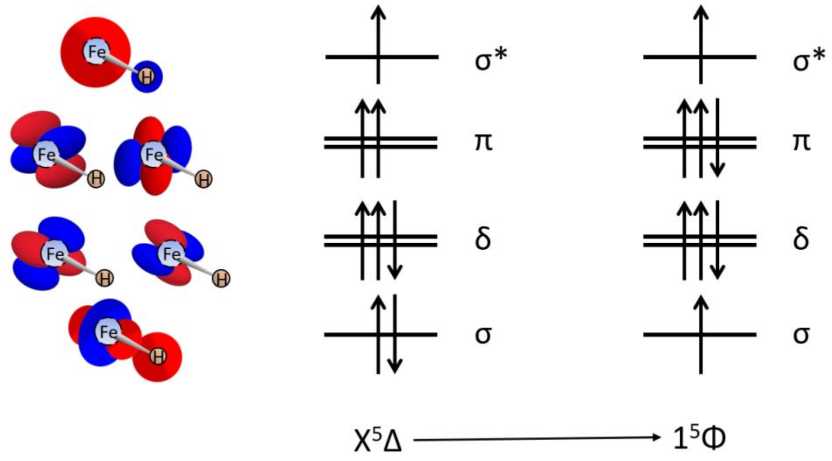

Figure S5. Qualitative representation of molecular orbitals of the $X^5\Delta$ ground state and the electronically excited state $1^5\Phi$. Molecular orbitals labeled π or δ are exactly degenerate.

Table S1. Parameters used for the PGOPHER fit in Figure S2 (b-d). Rotational constants B were obtained from Fig. 2 of the main text, initial guesses for spin-orbit coupling constants A were obtained from calculations.

$1^5\phi \leftarrow {}^5\Delta$	Ground state	Excited state
	adjusted	Adjusted
S	2	2
Λ	Δ	ϕ
Origin / cm^{-1}	0	19003
B / cm^{-1}	6.744	3.537
A^a / cm^{-1}	-99.9	-100.2
λ^b / cm^{-1}	-2.3	-2.1
γ^c / cm^{-1}	-8.7	-8.1

$1^5\Pi \leftarrow {}^5\Delta$	Ground state	Excited state
S	2	2
Λ	Δ	Π
Origin / cm^{-1}	0	18582
B / cm^{-1}	6.744	3.537
A^a / cm^{-1}	-98.7	-103.3
λ^b / cm^{-1}	-2.9	-2.9
γ^c / cm^{-1}	-21.1	-8.5

$1^5\Delta \leftarrow {}^5\Delta$	Ground state	Excited state
S	2	2
Λ	Δ	Δ
Origin / cm^{-1}	0	18838
B / cm^{-1}	6.744	3.520
A^a / cm^{-1}	-99.2	-99.9
λ^b / cm^{-1}	-3.8	-1.1
γ^c / cm^{-1}	-9.0	-20.6

[a]A : Spin-orbit coupling constant

[b]λ: Spin-spin coupling constant (LamdaSS in PGOPHER)

[c]γ: Spin- rotation coupling constant (Gamma in PGOPHER)

Table S2. AQC parameters are used for Figure 3b, $1^5\phi \leftarrow {}^5\Delta$ transition.

Empirical fit	Ground state		Excited state	
	modified	initial guess	modified	initial guess
S	2		2	
Λ	Δ		ϕ	
Origin / cm^{-1}	0		18854	
B / cm^{-1}	6.788	6.788	3.825	3.825
A^a / cm^{-1}	-90.8	-102.9	-95.2	-91.7
λ^b / cm^{-1}	-4.9	-2	29.5	+17
γ^c / cm^{-1}	-6.2	-2.1	-2.6	-1.2

[a]A: Spin-orbit coupling constant

[b]λ: Spin-spin coupling constant (LamdaSS in PGOPHER)

[c]γ: Spin- rotation coupling constant (Gamma in PGOPHER)

References

(1) Hobbs, L. M., York, D. G., Thorburn, J. A., Snow, T. P., Bishof, M., Friedman, S. D., McCall, B. J., Oka, T., Rachford, B., Sonnentrucker, P., Welty, D. E. Studies of the Diffuse Interstellar Bands. III. HD 183143. *Astrophys. J.* **2009**, *705*, 32–45.
